# Advances and Gaps in Global Newborn Screening for Sickle Cell Disease

**DOI:** 10.3390/ijns12010004

**Published:** 2026-01-21

**Authors:** Lisa Marie Shook, Russell E. Ware

**Affiliations:** 1 Division of Hematology, Department of Pediatrics, Cincinnati Children’s Hospital Medical Center, Cincinnati, OH 45229, USA; russell.ware@cchmc.org; 2 Comprehensive Sickle Cell Center, Cincinnati Children’s Hospital Medical Center, Cincinnati, OH 45229, USA; 3 Department of Pediatrics, College of Medicine, University of Cincinnati, Cincinnati, OH 45229, USA; 4 Global Health Center, Cincinnati Children’s Hospital Medical Center, Cincinnati, OH 45229, USA

**Keywords:** newborn screening, sickle cell disease, global health, health equity

## Abstract

Newborn screening (NBS) for sickle cell disease (SCD) has been performed in the United States (US) for decades, significantly reducing infant morbidity and mortality. A landmark clinical trial demonstrated that early identification of SCD enabled timely and life-saving prophylactic penicillin; this led to recommendations for universal NBS across the US. Early use of hydroxyurea as a safe and effective treatment for SCD further improved clinical outcomes by preventing acute and chronic disease complications. These advances add to the importance of early diagnosis through NBS, providing an opportunity for early treatment intervention. In recent years, high-resource countries—including those in Europe, the UK, and Canada—have adopted NBS for SCD using diverse strategies. Simultaneously, pilot programs in lower-resource settings such as Africa, Brazil, and India have demonstrated local feasibility and impact through implementation efforts. An overarching equity gap for achieving global NBS for SCD is the variable access to simple, accurate, and affordable testing. Other challenges include timing of NBS testing, targeted populations, laboratory methods, and parental education with genetic counseling. Questions remain about the equitable enrollment of affected infants worldwide into comprehensive care to ensure early treatment. These challenges raise concerns about sustainability, underscore the need for long-term funding and a strategic plan, and highlight persistent inequities from the lack of global NBS standards.

## 1. Newborn Screening for Hemoglobinopathies in the United States

After developing the innovative concept of screening neonatal biological samples for the early diagnosis of a congenital disease (phenylketonuria using neonatal urine specimens) [1], Dr. Guthrie published a seminal article in 1973 that established sickle cell disease (SCD) as another inherited disorder suitable for early detection [2]. Using blood spots on filter paper, his team documented that SCD could be accurately diagnosed, thereby allowing early identification of affected infants.

At almost the same time, SCD became recognized in the United States (US) through the efforts of then-President Nixon, who highlighted the disorder and called for increased research and treatment, which led to the passage of the National Sickle Cell Anemia Control Act of 1972. Soon after, newborn screening (NBS) for the early identification of SCD was established in the US, beginning in New York and then slowly expanded to other states [3].

In 1986, a landmark clinical trial was published, *Prophylactic Penicillin Study: A Randomized Trial of Penicillin Prophylaxis in Children with Sickle Cell Anemia* (PROPS), which demonstrated that twice-daily oral prophylactic penicillin was a life-saving intervention for young patients with SCD. The prevention of fatal infections provided the rationale for early screening, and a 1987 National Institutes of Health Consensus Conference recommended SCD screening for all newborns in all states across the US to allow early initiation of penicillin prophylaxis [4].

Expansion of NBS for SCD was phased in gradually across the US, with most states mandating universal newborn screening between 1987 and 1993. By 1990, roughly half of the US had adopted mandatory screening laws, but the last state finally adopted universal newborn screening in 2006 [5]. SCD was included in the first Recommended Uniform Screening Panel (RUSP) created by the Advisory Committee on Heritable Disorders in Newborns and Children [6]. Currently, all states and territories identify newborns with SCD and have formal programs for retrieval and retesting before enrolling in care and management. Considerable variation exists, however, in access to treatment across the US.

Laboratory testing for hemoglobinopathies in the United States has included a variety of sophisticated methods such as cellulose acetate electrophoresis, tandem mass spectrometry, isoelectric focusing, and high-performance liquid chromatography [7]. However, the resources necessary for these testing methods can be cost-prohibitive in lower-resource countries, which significantly limits the ability to scale up these methods for widespread newborn screening programs.

Life expectancy for SCD is still substantially shortened in the US, despite advances in care and an increasing life expectancy. In the 1970s, one of the first natural history studies for SCD estimated that the life expectancy was under 20 years old for individuals with SCD [8]; however, after NBS was implemented, a study in the 1990s found that survival had extended to a median life expectancy of 42 years old for men and 48 years for women [9]. Recently, a large cohort study estimated a life expectancy of ~54 years for individuals with SCD compared to 76 years for those individuals without SCD [10]. This increase in life expectancy can be credited to the implementation of interventions, including newborn screening, penicillin prophylaxis, and recent treatment advances, which may be scalable to low-resource settings as well.

## 2. Global Expansion of Newborn Screening for Hemoglobinopathies

Approximately 90% of worldwide SCD births occur in regions of the world where NBS programs have not been widely implemented, particularly Sub-Saharan Africa and India, where barriers such as logistical operations and the prohibitive cost of newborn screening have prevented rapid expansion [11]. In West Africa, SCD is one of the leading causes of childhood mortality, with approximately 43% of children dying by age 10 because of the lack of NBS diagnosis and treatment [12]. However, no country in Africa has moved beyond the pilot stage toward implementing universal NBS for SCD [13].

Global efforts to address this overwhelming disparity have been urged by the United Nations General Assembly, which declared SCD a disease of public concern, as well as the World Health Organization (WHO), which declared SCD a priority non-communicable disease [14,15]. The WHO estimates that nearly 70% of existing SCD mortality could be prevented with the implementation of early diagnosis and intervention programs. While NBS has been proven to be effective for early diagnosis and treatment to reduce mortality and morbidity in high-resource countries, there are still concerns about implementing newborn screening in low-resourced countries, where the implementation of universal NBS is hindered by a lack of sustainable funding, lack of laboratory infrastructure, and the overall cost of diagnosis, as well as the challenges of locating and educating families about abnormal results in a timely manner to initiate treatment [15].

Over the past fifteen years, both the United Nations and the World Health Organization (WHO) have identified SCD as a global health burden, with hemoglobinopathies found in 71% of 229 countries around the world [16]. In 2010, the WHO created a policy framework for SCD with the main targets including establishing national SCD control programs in all high-prevalence countries; reducing child mortality due to SCD; integrating SCD services into existing maternal and child health programs, particularly at the primary care level; ensuring access to essential treatments such as folic acid and penicillin; improving public and professional awareness about SCD; developing surveillance and registry systems to monitor disease prevalence and program outcomes; and promoting innovative research [17].

India is estimated to be one of the top three countries in the world most affected by SCD, and it is estimated that NBS could save over 10 million children’s lives by 2050 [18]. There is currently no standardized country-wide NBS program, however, and children with SCD are typically tested and confirmed as symptoms and complications manifest. There have been several regional newborn screening programs that have been implemented, primarily in tribal populations that reside in remote regions far from major cities [19]. Sample methodologies used in India include cord blood, dried blood spots tested with HPLC-variant testing systems, and a point-of-care rapid test SICKLE-CHECK^TM^, which is a semi-reliable solubility test that is similar to SICKLE Dex [20]. HPLC technology was used because the machines were already accessible for other programs, and so testing did not require new infrastructure or equipment, and the machines are easily operated in rural areas [21]. As a result of some of the remote region pilot programs, the Ministry of Health and Family Welfare crafted Guidelines for a National Hemoglobinopathy Program and launched the “National Sickle Cell Anaemia Elimination Mission”, which includes NBS for SCD with components for prevention, management, operational support, and policy change to improve care of SCD patients and lower the prevalence by 2047 [22]. There is a live public dashboard for the National Sickle Cell Anaemia Elimination Mission that shows screened numbers, including counts of trait, negative, and disease results, and “today’s result”. To date, over 60 million people have been tested [23].

In Europe, there are ongoing efforts to develop consensus and support the development of NBS programs across the continent. In 2017, a two-day Pan European consensus conference summarized the current status of NBS for SCD and developed consensus-based indications and screening methodology in Europe. Two approaches to NBS for SCD were proposed: “universal screening”, which is offered to the entire newborn population, or “targeted screening”, which considers the ethnic ancestry and restricts testing to babies whose family origins are from “at risk” ethnic populations [24]. The rationale is that European countries with a predominantly “white” population can focus testing on immigrant populations at the highest risk for SCD; however, this approach will be problematic after several generations, when the genetic risk has spread across the population and targeted screening will miss affected infants. Several European countries and the United Kingdom have universal newborn screening [25]. However, some countries, such as those in the Mediterranean regions of Europe, have focused on expanding prenatal testing, but the effectiveness of this approach is still largely unknown [26].

In South America, the Brazilian Ministry of Health has implemented a National Neonatal Screening Program (NNSP) for all 26 states throughout the country, which has been universally screening since 2013 for sickle cell disease and other hemoglobinopathies. With a population of over 200 million people, the incidence of SCD in regions of Brazil has ranged from 1:650 births to 1:13,600 births. The implementation process has taken over 40 years, from the initial planning at the local grassroots level in the 1980s to creating recent achievements, including the Policy of Integral Attention to People with Sickle Cell Disease [27]. The goals of this new policy are to provide genetic guidance, safeguard the reproductive rights of those with sickle cell trait, and to educate the general population about SCD. A Technical Advisory Council for Sickle Cell Disease was established by the Ministry of Health in Brazil, composed of healthcare specialists, educational, research, and assistance organizations involved in the care of people with SCD, managers of municipal and state jurisdictions, and healthcare specialists. This led to the development of best practices and educational materials about the management of SCD and its complications, genetic and family education, and the role of nursing. Plans were put into place to provide basic care with early identification of SCD through the NNSP to provide free prophylaxis with penicillin and referral to a regional specialized care center, where families can also receive healthcare—including hydroxyurea and transcranial Doppler examinations—to identify stroke risk [27].

In the Caribbean, the estimated incidence of SCD overall is estimated to be 2000–3000 infants born annually with SCD, with high variance across islands such as Jamaica (1:150), Grenada (1:275), and Guadeloupe and Martinique French territories (combined prevalence 1:300). As a result, the Caribbean area has created a regional clinician–researcher network (CAREST) to coordinate capacity-building, epidemiology, advocacy, and the implementation of newborn screening and early treatment intervention across English-speaking Caribbean islands and French overseas territories (e.g., Martinique and Guadeloupe), along with international partners to address equity gaps in data, lab capacity, and follow-up care [28]. This partnership has expanded NBS follow-up across several islands, beginning with publishing prevalence estimates and accelerating the use of rapid/point-of-care screening approaches. The Caribbean efforts have encountered challenges, including small populations across culturally and geographically dispersed islands, resulting in logistical considerations to transport specimens for testing. In addition, there are variable health infrastructures both across islands and within areas of islands that lead to inconsistent follow-up of newborns identified with SCD [28]. Countries such as Jamaica, Guadeloupe, Cuba, and Martinique have established NBS programs, while pilot programs have been launched in Barbados, St. Lucia, Grenada, Trinidad, Tobago, and the Bahamas. The NBS program has proven to be impactful in Jamaica, with a reduction in childhood mortality from SCD from 30% to less than 5% in screened populations [29].

In the Middle East, newborn screening for SCD is largely dependent on financial stability. The Gulf states, such as Saudia Arabia, United Arab Emirates (UAE), Bahrain, and Oman, are often well-funded and have strong healthcare systems with the ability to meet the needs of NBS, while conflict-affected regions such as Yemen, Syria, and Iraq are much less successful, as basic health needs such as vaccinations and maternal/child healthcare are prioritized [30]. NBS in these latter regions are often limited to pilot projects or are nonexistent; infrastructure, personnel, and supply changes are major barriers to implementation, as well as the funding gaps and political turbulence. The UAE leads with near-universal coverage (95% of the population), achieved in 2010 due to a long-standing national screening program [30]. Saudi Arabia, among other countries, is continuing to expand coverage to reach over 85% of the population, after adding hemoglobinopathies to its NBS panel in 2023 [31]. Other countries, such as Bahrain (2.1% SCD prevalence) [32] and Oman (4% SCD prevalence) [33], have moderate NBS coverage, but it can be variable and regionally dependent, despite having some of the highest SCD prevalence rates in the Middle East. In Lebanon, a study from 2010 to 2013 showed that the incidence of SCD and other hemoglobinopathies was 21 per 1000 newborns, and the majority were non-Lebanese in origin—most likely the result of migration from Africa and other Middle Eastern countries [34]. Expanding NBS to other Middle Eastern countries is dependent on building infrastructure and training personnel, along with cost and political instability.

In Sub-Saharan Africa, there have been many pilot NBS programs, all documenting the general feasibility of testing for SCD and a high prevalence of both trait and disease. However, to date, none has expanded beyond a local effort toward a national testing platform. In West Africa, results have been reported from Benin, the Gambia, Ghana, Guinea Bissau, Liberia, and Mali [35,36,37,38,39,40]. In Middle Africa, screening data have been published from Angola, the Democratic Republic of Congo, Nigeria, and Zambia [41,42,43,44]. Eastern Africa data are available for Kenya, Malawi, Tanzania, and Uganda [45,46,47,48]. Since Sub-Saharan Africa is where the largest number of annual SCD births occur, efforts to improve NBS across the continent will potentially have the greatest impact.

## 3. Testing Methodology

Newborn screening for SCD employs a range of diagnostic laboratory methodologies, each with distinct implications for accessibility, accuracy, and equity. These methods vary in complexity, cost, and scalability, which can influence how they can be implemented equitably across diverse populations and geographical settings. There are several equity considerations—including infrastructure and access, cost and sustainability, global disparities, and ethical and cultural sensitivity—to consider with laboratory methodology. As NBS for SCD has expanded globally, the methodology for screening has improved, but each technique has its own pros and cons for implementation and varies widely in cost and sensitivity.

In many countries, including the US, neonatal blood spot collection by a heel stick is tested using isoelectric focusing (IEF) or high-performance liquid chromatography (HPLC). IEF allows high throughput, is widely validated, and can help identify hemoglobin variants, but it requires expensive equipment, batch processing, and has limited portability. This method is most often found in centralized labs in high-resource countries such as the US and across Europe. In comparison, HPLC is accurate and can be automated to assist throughput, with the ability to identify hemoglobin variants. However, HPLC requires expensive equipment, as it is not portable and requires frequent maintenance. This methodology works well in high-resource countries and reference laboratories [49].

Other testing methodology includes capillary electrophoresis (CE), which is recommended for confirmatory testing because of its high level of accuracy and automated system; this method is used in some labs in the US and across Europe [50]. Tandem Mass Spectrometry (MS/MS) is a highly sensitive methodology that is highly accurate [51] and used successfully in many European countries [52,53,54,55]. An additional advantage of mass spectrometry is the ability to screen for multiple genetic disorders at once, as currently implemented in Europe and the UK [56,57,58]. DNA and molecular testing are being piloted with programs in the US, Europe, and globally. Its clinical utility lies in its simplicity and accuracy, as well as the elimination of false positives with highly specific variant diagnoses [59,60], and will be helpful in the gene therapy era since these labs can provide clinical data that protein-level or HPLC-based tests cannot capture. A disadvantage to this testing method is that it can have a slower turnaround time and higher costs for equipment and testing.

Key recent advances in testing methodology include the development of point-of-care tests for accurate and reliable NBS for SCD, which is especially useful for low-resource countries. Point-of-care tests, such as Gazelle^TM^, SickleSCAN^TM^, and others, can be implemented in lower–middle-income countries with minimal infrastructure. The Gazelle is a single-use and semi-quantitative microchip electrophoresis technique that is designed to detect hemoglobin A and common variants such as hemoglobin S, hemoglobin C, and hemoglobin E; recent improvements also allow quantitation of hemoglobin F [61]. The SickleSCAN^TM^ technique is a lateral-flow immunoassay, and until recently has been non-quantitative. These tests provide rapid results (often under 10 min), and the equipment is highly portable [62]. These low-cost methodologies are being piloted and implemented in areas such as Sub-Saharan Africa, India, Haiti, and other rural areas globally. All positive results using POC devices should be confirmed with a second test, and the results documented in the medical records to prevent repeat testing.

## 4. Treatment Advances

Hydroxyurea as a safe and effective disease-modifying therapy for SCD has further improved clinical outcomes and reduced early organ damage in infants over the past 30 years. In 1995, the results of the *Multicenter Study of Hydroxyurea in Sickle Cell Anemia* were published and provided evidence that hydroxyurea is efficacious for reducing vaso-occlusive crises [63]. This trial led to hydroxyurea being approved as the first drug authorized for SCD by the Food and Drug Administration in 1998. Since then, more than 100 interventional trials with hydroxyurea have continued to demonstrate its clinical benefits, including the following: reduced hospitalizations and need for blood transfusions, delay or prevention of organ damage, and reduced mortality. Studies such as the BABY HUG trial demonstrated safety early in life, leading to the FDA approving treatment for children as young as 9 months. This has also led to hydroxyurea becoming a standard treatment for SCD and the benchmark therapy against which new treatments for SCD must be compared [64].

While there are clear clinical benefits to hydroxyurea, global clinical research and implementation of this potent disease-modifying therapy have been challenging. It is noteworthy that many of the hydroxyurea studies were conducted in the United States, Europe, and Brazil, while the majority of individuals living with SCD are located in low-resource settings like India and Sub-Saharan Africa. Moreover, although hydroxyurea is considered reasonable in cost by some standards (often under USD 1 a day in most countries), that amount can be a limiting factor to access in many areas of the world. Estimates in Sub-Saharan Africa are that only 1–13% of individuals with SCD receive hydroxyurea on a regular basis [64]. There are also other geographic consequences to consider with treatment, including the clinical research question in Africa about the possible unintended consequence of worsening the severity of malaria, which was disproven by the NOHARM trial (Novel use Of Hydroxyurea in African Regions with Malaria) trial [65], and found no increase in malaria; subsequent larger trial including REACH documented that hydroxyurea actually reduces the severity and frequency of malaria [66].

In the US, the TWiTCH (Transcranial Doppler with Transfusion Changing to Hydroxyurea) study found that hydroxyurea was a safe and highly effective alternative to the usual treatment of chronic blood transfusions for children with elevated transcranial Doppler (TCD) velocities [67]. This was an important clinical discovery as blood transfusions can be logistically challenging and costly, and limited in low-resource settings, such as Sub-Saharan Africa. Subsequently, the effectiveness of hydroxyurea for preventing primary and secondary stroke in SCD has been demonstrated in low-resource settings, including Jamaica (EXTEND trial) [68], Tanzania (SPHERE trial) [69], and the Dominican Republic (SACRED trial) [70].

Despite bone marrow transplant being considered a curative therapy for SCD, global access to this remains highly inequitable, as this treatment is significantly limited to high-income countries. Disparities in bone marrow transplantation include the high costs of transplantation and limited access to matched donors [71]. While research is currently exploring different approaches to stem cell therapy, innovative strategies that consider capacity building, regional transplant centers, donor registry diversity, and follow-up support services are necessary to translate this therapy into a real-world benefit worldwide [72].

In 2023, the FDA approved two groundbreaking gene therapy treatments for SCD—Casgevy and Lyfgenia [73]. However, this advancement comes with several barriers, including coverage of the cost of treatment, which averages between USD 2–3 million just for the product and requires significant costs for the procedure, including follow-up care and support services. There is concern about how this treatment could ever be expanded globally, especially in high-burden areas such as India and Africa, as there currently is no infrastructure to support transplant and cell-processing facilities, or regulatory capacity, sustainability for the cost of treatment, and support for long-term follow-up. There is concern that these inequities may continue to narrow the global treatment gap and further reduce access to innovative treatments [74].

## 5. Implications

An important global challenge for SCD screening is the lack of a uniform testing approach to ensure all babies are accurately screened at birth. Several of the laboratory methods that identify SCD have limitations in the newborn period due to the small amount of sickle hemoglobin present at birth. In addition, although a consensus conference in Europe recommended considering universal screening, the implementation of such an undertaking that focuses on provider education, standardization of screening protocols, integration with National Health Systems, data collection and monitoring, and equity in access has proven difficult, and instead, there has been a focus on targeted screening of recent immigrants [24].

In many low-resource settings, such as Africa, babies are not always born in a traditional hospital setting, which can be addressed by screening infants at established immunization centers during the first few months of life. Additional challenges include barriers to transportation of newborn screening samples, babies with positive screening results not being located reliably, lack of trained healthcare providers, and cost-prohibitive lab reagents, equipment, and electricity [75].

A broader challenge is the long-term follow-up of newborns successfully identified with SCD through screening. In the US, a survey of 39 state newborn screening programs found that 12 did not provide any long-term follow-up after the diagnosis of SCD. Moreover, only 18% of children aged 3 months to 5 years received the recommended 300+ days of antibiotic prophylaxis, and more than 25% lacked documentation of pneumococcal vaccine [76].

These challenges highlight critical gaps and broad health system inequities in the continuum of care for children diagnosed with SCD through newborn screening. Moreover, the global challenges point to several opportunities for policy reform, quality improvement, and implementation strategies to improve access to screening as well as care.

## 6. Strategies Toward Global Access to Newborn Screening

In recent years, there have been efforts to expand global access to newborn screening for SCD. The American Society of Hematology (ASH) established CONSA (Consortium on Newborn Screening in Africa) to focus on scaling up NBS in Sub-Saharan Africa for early diagnosis and ensure access and linkages to comprehensive care, which will reduce under-five mortality among infants with SCD. CONSA is focused on seven countries (Uganda, Zambia, Ghana, Kenya, Liberia, Nigeria, and Tanzania), and has recently reported initial screening results of 175,000 infants [77]. CONSA is creating a framework of comprehensive care programs that include access to penicillin prophylaxis, immunization, and best practices for follow-up care for SCD, and also building local capacity—including training laboratory and clinical staff, establishing labs for processing dried blood-spot screening, and implementing quality-control standards and specimen transport strategies. Data from CONSA will be tracked in a data hub managed by ASH in order to demonstrate how effective coordinated international-local efforts can bridge gaps in NBS for SCD in Africa [78].

Another example of global efforts is the ARISE Project (African Research and Innovative Initiative for Sickle cell Education), which is based in London with involvement from institutions in Italy, France, Cyprus, Nigeria, Lebanon, Kenya, Angola, Zambia, and the US. The goal of ARISE is to create an interagency and multidisciplinary staff exchange program between early investigators, researchers, and other staff members to share and spread best practices in the diagnosis and treatment of SCD with NBS. Some of the strategies include implementing eHealth technologies for NBS in Nigeria, Kenya, and Lebanon; improving laboratory diagnostics and quality assurance; and improving the management of SCD through molecular diagnostic techniques and genetic counseling [79]. ARISE was concluded in October 2024, having met all objectives and milestones, including the training of fellows.

One possible high-impact strategy to accelerate improvements in global access to newborn screening is utilize existing frameworks that have been successful, such as the GAVI Vaccine Alliance’s approach to implementation. This model was developed as a result of the inability to rapidly spread new vaccines to the poorest developing countries to improve global immunization rates in the 1990s. GAVI, established in 2000, created a public–private alliance model that includes the World Health Organization, the Gates Foundation, UNICEF, the World Bank, governments, scientific institutions, and civil society institutions to increase access to life-saving vaccines. Funding was the cornerstone of effective implementation that included delivery system improvement, educating healthcare professionals, and designing surveillance programs while focusing on supply chain management and cost-effectiveness across 73 countries [80]. Engaging drug companies using GAVI’s model could be effective in spreading access to hydroxyurea globally. Costs could be negotiated to more affordable rates, and perhaps co-financing could be offered to GAVI-eligible countries with the goal of becoming self-sustaining.

The lack of global access to NBS for SCD presents several opportunities, including policy, quality improvement, and implementation strategies [81]. Policy opportunities include standardizing care protocols, including newborn screening follow-up, prophylactic penicillin, and vaccinations. Quality improvement can be an opportunity to improve data collection and reporting, including the development of electronic health records and registries to help track and improve care for newborns, as well as developing performance indicators to track quality metrics, such as the % of newborns screened; % of newborns diagnosed with SCD that also receive antibiotic prophylaxis and documented vaccinations; and % of newborns receiving disease-modifying therapy, such as hydroxyurea [82]. Implementation strategies include partnering with private funders and governments for sustainability, testing telehealth expansion, and developing healthcare provider capacity by increasing evidence-based knowledge about SCD with training and education.

## 7. Discussion

NBS for SCD has emerged as one of the most impactful public health interventions for reducing early childhood morbidity and mortality. However, despite decades of evidence and technological progress, the global implementation of NBS for SCD remains highly inequitable. The majority of infants in the world born with SCD still lack access to timely diagnosis and life-saving interventions. Alarmingly, this is not due to a gap in scientific knowledge but instead is a systemic failure to translate knowledge into practice equitably.

This lack of equity presents an opportunity for the global newborn screening community to create solutions. Strategies can be developed to ensure that all newborns, regardless of socioeconomic status or geography, have access to reliable screening methods and that follow-up can be a defining goal to reduce mortality. Particularly in regions of the world with the greatest burden, universal newborn screening for SCD should be framed as a human right that every child has the opportunity to live a healthy life, regardless of geography, ethnicity, or income, and should not be a reflection of privilege in wealthier nations.

Strategies for consideration that may guide improving and standardizing global NBS efforts include the following:

- Integration with Maternal and Child Health Systems: Embed NBS for SCD into existing infrastructures to improve continuity of care and enable sustainability without having to create health systems or frameworks. Community health workers can also be invaluable in educating families and building trust in the healthcare system.
- Capacity Building and Workforce Development: Healthcare providers, lab personnel, and community health workers can be the key to ensuring screening is high-quality and diagnosis is timely, while providing culturally competent care to all.
- Access to Treatment: Screening without access to care is an empty promise and is likely worse for low-resource countries than not screening at all. Screening is just the first step and must be accompanied with access to preventative as well as disease-modifying therapies such as penicillin prophylaxis, immunizations, and hydroxyurea. Moreover, equitable access to future curative therapies, such as bone marrow transplant and gene therapy, should be explored to avoid further deepening the global treatment chasm.
- Data Systems and Quality Improvement Metrics: Continuous improvement tracked by establishing registries and performance indicators, such as screening coverage, linkages to follow-up care, and treatment initiation for newborns, can not only lead to better outcomes but also track equitable access.
- Sustainable Financing Models: Leveraging public–private partnerships and global health mechanisms, such as those developed by the GAVI Vaccine Alliance and CONSA, can help support cost-effective implementation in low-resource settings and lead to scalable efforts. The successful models have demonstrated shared risk, coordinated investments, and strategic partnerships to overcome cost barriers.
- Innovation: There is an opportunity to further expand and develop advances in point-of-care diagnostics with cloud-based access to results, mobile health technologies, and leveraging lab networks to continue to expand NBS capacity in limited-resource settings. However, innovation should be equity-driven and designed for accessibility.
- Equity-Driven Policy and Standards: The lack of international consensus on best techniques, minimum standards for NBS screening, and follow-up and treatment for SCD that is adapted to local, geographical, and cultural contexts is further increasing the global gaps. Considering all the different health systems and barriers is necessary for beginning to approach an international consensus. However, “one size does not fit all”, so the consensus needs to consider different approaches that would be adaptable to local contexts but anchored in equity [83].

There is also the opportunity to further explore digital health innovations to support SCD screening. For example, as infrastructure and capacity are built, using technology such as mobile health platforms with SMS-based texting systems could track screening outcomes and send reminders for follow-up care, especially in areas with limited internet access. Electronic health records can also improve continuity of care for patients seen in multiple locations, and can enable centralized data collection for public health monitoring. Telemedicine may also emerge to allow hematologists to guide diagnosis and treatment in areas that are lacking providers. Finally, cloud-based data systems can be invaluable to enable real-time reporting of NBS data and help governments and other stakeholders create registries and monitor program effectiveness.

The essence of health equity for NBS and SCD is the devastating paradox that the highest burden of disease exists where the fewest resources are available to address it. The path forward is clear—equity must be the lens through which every decision about NBS for SCD is made. This is a moral challenge for global health leaders, researchers, clinicians, and advocates. Systems must be built to reflect inclusion and justice. This is the only way to ensure that NBS can reach every child, in every corner of the world, not just to save lives but also transform them sustainably and equitably.

## Data Availability

No new data were created.

## Abbreviations

The following abbreviations are used in this manuscript:

ARISE	African Research and Innovative Initiative for Sickle Cell Education
BABYHUG	Hydroxyurea in Very Young Children with Sickle Cell Anemia
CAREST	Caribbean Network of Researchers on Sickle Cell Disease and Thalassemia
CE	Capillary electrophoresis
CONSA	Consortium on Newborn Screening in Africa
EXTEND	Expanding Treatment for Existing Neurological Disease
FDA	Food and Drug Administration
GAVI	Global Alliance for Vaccines and Immunization
HPLC	High-performance liquid chromatography
IEF	Isoelectric focusing
MS/MS	Tandem mass spectrometry
NBS	Newborn screening
NNSP	National Neonatal Screening Program
NOHARM	Novel use of Hydroxyurea in African Regions with Malaria
POC	Point-of-care
PROPS	Penicillin Prophylaxis in Children with Sickle Cell Anemia
RUSP	Recommended Uniform Screening Panel
SACRED	Stroke Avoidance for Children in Republica Dominicana
SCD	Sickle cell disease
SPHERE	Stroke Prevention with Hydroxyurea Enabled Through Research and Education
TCD	Transcranial Doppler
TWiTCH	Transcranial Doppler with Transfusion Changing to Hydroxyurea
UAE	United Arab Emirates
US	United States
WHO	World Health Organization
